# Sealing efficacy of mineral trioxide aggregate with and without nanosilver for root end filling: An *in vitro* bacterial leakage study

**DOI:** 10.4317/jced.53070

**Published:** 2017-01-01

**Authors:** Mahsa Eskandarinezhad, Naiemeh Shahveghar-Asl, Reza Sharghi, Sajjad Shirazi, Sahar Shakouie, Amin-Salem Milani, Esrafil Balaei

**Affiliations:** 1 Assistant professor, Department of Endodontics, Faculty of Dentistry, Tabriz University of Medical Sciences, Tabriz, Iran; 2 Post-graduate student, Department of Pediatric Dentistry, Faculty of Dentistry, Qazvin University of Medical Sciences, Qazvin, Iran; 3 Assistant professor, Dental Caries Prevention Research Center, Qazvin University of Medical Sciences, Qazvin, Iran; 4 Research Fellow and Lecturer, Dental and Periodontal Research Center, Faculty of Dentistry, Tabriz University of Medical Sciences, Tabriz, Iran; 5 Assistant Professor, Department of Community Dentistry, Faculty of Dentistry, Tabriz University of Medical Sciences, Tabriz, Iran

## Abstract

**Background:**

Various materials have been added to mineral trioxide aggregate to enhance its properties. This study was aimed to compare the sealing efficacy of MTA with and without nanosilver using bacterial leakage approach.

**Material and Methods:**

Seventy canine teeth were prepared and obturated. Then, after apical resection, the root-end cavities were prepared by ultrasonic retrotips. Teeth were randomly divided into 4 groups containing two experimental groups (n=30) and two negative and positive controls (n=5). In group 1 and 2, root-end cavities were respectively filled with MTA and MTA with nanosilver (by 1% weight). Leakage assessment was carried out by bacterial leakage apparatus with *Enterococcus faecalis* species. Leakage comparison between experimental groups was done using Mann-Whitney test by Spss 16 software at significancy level of 0.05.

**Results:**

The median bacterial leakages for MTA and MTA with nanosilver were 19 and 2, respectively. The mean bacterial leakages for MTA and MTA with nanosilver were 30.06±28.67 and 9.66±14.25, respectively. Mann-Whitney test indicated that there was a significant difference in bacterial leakage day between two experimental groups (*P*=0.002).

**Conclusions:**

Based on the findings of this *in-vitro* bacterial leakage study, adding nanosilver to MTA decreased its sealing ability.

** Key words:**Root canal therapy, root canal obturation, root canal filling materials, nanosilver, MTA.

## Introduction

Endodontic failures are almost associated with incomplete cleaning of root canal system and consequently penetration of bacteria and other pathogens into the periradicular tissues (1). Therefore, root-end filling materials should have antimicrobial activity to inhibit microbial growth in addition to appropriate sealing ability and biocompatibility (1). Various materials such as glass ionomer, Super-EBA, amalgam, composite, and recently mineral trioxide aggregate (MTA) have been utilized for filling root-end cavities (2). Studies regarding the sealing ability of MTA have depicted its superior sealing ability in comparison to other materials (2).

For a long time silver has been known to have a disinfecting effect and its salts and their derivatives are commercially employed as antimicrobial agents (3). Silver nanoparticles (AgNPs or nanosilver) are one of the most widely used nanoparticles, for antimicrobial purpose in medical applications (4).

The antimicrobial effect of nanosilver might be explained by the interaction of nanoparticles with microbes involving silver ion release and particle cellular internalization (5). Size-dependent toxicity of nanosilver supports the mode of action of nanosilver. The nanosilver toxicity is species-specific. Small-sized nanosilver can inhibit nitrifying bacterial growth more than silver ions at the same total silver concentration (6). Samiei *et al.* (7) reported that adding nanosilver by 1% weight to MTA improved its anti-microbial activity.

Based on the previous studies which depicted desirable properties of the MTA and nanosilver mixture, this study was aimed to evaluate the sealing efficacy of MTA with and without nanosilver in root end cavities prepared by ultrasonic.

Different methods have been employed for assessment of leakage. Many problems related to dye application have been discussed. Dye leakage did not have the required standard and have poor reproducibility (8). Although fluid filtration bear greater accuracy, clinical similarity of the bacterial leakage method is more than fluid filtration (9); therefore bacterial leakage was used in this study.

## Material and Methods

The protocol for this study was independently reviewed and approved by Institutional Review Board of the university (Ref No. 2438). According to the study performed by Erkut *et al.* (10), and considering α equal to 0.05 and power of 80% (11) with 15 % difference in microleakage, twenty six samples in each group was needed. However, thirty samples per each group were used to increase the power of study. In addition, ten other teeth were used as positive and negative controls. Seventy human single-canal canine teeth extracted for orthodontic or periodontal purposes were selected for this study. Teeth were examined by stereomicroscope (Olympus SZ, 9-ILL B200-Choida KU, Japan) and radiographs. The roots with cracks, caries, external or internal resorption, and canal pathway calcification were excluded 

-Preparation and filling of root-end cavities

The teeth were cleaned of extraneous tissue and calculus, and then immersed in 5.25% NaOCI (sodium hypochlorite) for 5 minutes and were stored in PBS (phosphate buffer solution) to prevent dehydration. To gain a straight-forward access to root canal system, all specimens were resected at 16mm from the apices using a water-cooled diamond disk (D&Z, Darmstadt, Germany). All procedures were performed by a single operator. Working length was determined by inserting a #15 K-file (Mani, Nakanishi Inc., Tokyo, Japan) into the canal until it was just visible at the apical foramen, then subtracting 1 mm. Root canals were prepared using ProTaper rotary Ni-Ti instruments (Dentsply Maillefer, Ballaigues, Switzerland) on an electrical endodontic handpiece (TCM Endo III, Sybron Endo, USA) at 250 rpm. Preparation was carried out according to the manufacturer’s recommendations and crown-down technique. Briefly, the S1 file was used to clean and shape the coronal part of the canal. Subsequently, the SX file was used to increase the taper of the coronal region and S1, S2, F1, F2 and F3 were used sequentially to full working length. 15% EDTA gel (Glyde; Dentsply Maillefer) was used as a chelating agent and was delivered into the canal on the tip of each successive instrument. The canals were irrigated between instrumentations with 5 mL of freshly prepared solution of 2.5% NaOCl. Following instrumentation, the smear layer was removed with 15% EDTA, followed by 2.5% NaOCl. The canals were then dried with sterile paper points and obturated with gutta-percha (Ariadent, Tehran, Iran) and AH-26 sealer (Dentsply, Konstanz, Germany) using cold lateral condensation method. The teeth were stored at 37 ± 1°C and 100% relative humidity for 2 days. 

The apical 3 mm of each tooth was resected perpendicular to the long axis of the tooth with a diamond bur under continuous water and air spray. Root-end cavities were prepared to a depth of 3mm perpendicular to the long axis using ultrasonic retrotips Kis-3D (Spartan, Missouri, USA). A periodontal probe was used for depth measurement and then cavities were rinsed by normal saline and dried by paper cones. The teeth were randomly divided into 4 groups containing two experimental groups (n=30) and two negative and positive controls (n=5).

In group 1, root-end cavities were filled with white MTA (Angelus, Londrina, Brazil) which was manipulated according to the manufacturer’s instructions and inserted in cavities by MTA carrier and packed with a pluger. The material surface was burnished to remove the excess material. In group 2, the cavities were filled by MTA with nanosilver using the same procedures as group 1 was performed except, cavities were filled by MTA with nanosilver. Nanosilver (Silver Nano-powder 7440-22-4, Sigma Aldrich, USA) was added to MTA powder by 1% weight by a digital weighing machine (AND GR-200 Analytical Balance, Lab Recyclers Inc., Gaithersburg MD, USA) and then mixed with distilled water and inserted in cavities. In positive control group, the canal system was remained open. In negative control group, same procedure as group 1 was carried out. Teeth were kept at 37 ± 1°C and 100% relative humidity for 48 hours. In all specimens except for negative control group, external root surface except for apical and coronal portions were covered by two layers of nail varnish. In negative control group, all parts of specimens were covered by two layers of nail varnish.

-Bacterial leakage

**Figure 1:**
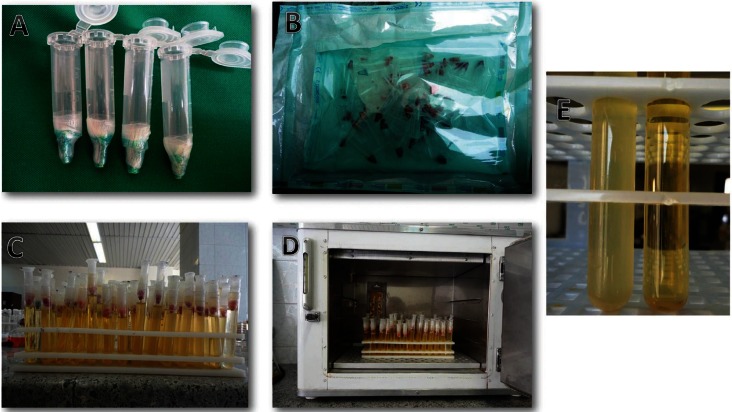
A) Tooth mounted to microtube. B) Sterilled and packed samples using ethylene oxide method. C) Apparatus used for bacterial leakage test (the upper chamber for microbial suspension and the lower one for BHI medium). D) Incubation of samples. E) Turbidity of growth medium.

For the evaluation of bacterial leakage and sealing ability, apparatus similar to that which was used in double-room technique (12) was developed and used in the present study. This apparatus was based on the straight fitting of two tubes: a 2ml centrifugation microtube and laboratory glass. The prepared and obturated teeth were fixed to the poly ethylene (Fig. 1A) microtubes and their interface was sealed by nail varnish and parafilm in order to make them impermeable, but the apical areas of the teeth were left free of varnish. In order to avoid contamination, the apparatus was sterilized using ethylene oxide gas (Fig. 1B) before mounting to glass. Mounting process was performed under microbial hood and sterile gloves. Then the microtube was inserted within glass, creating two separate chambers (Fig. 1C). In the lower one, 2mm of the root apex was remained immersed in the culture medium. In upper chamber the bacterial suspension was applied. The interface of microtube and glass was sealed by previously sterilized parafilm to provide lateral impermeability. The apparatus was developed to have a single pathway between the upper (microbial reservoir) and the lower chamber (culture medium), which allowed access to the root canal. This model would permit an assessment of any microbial microleakage that might occur through the root canal sealers.

-Microbial preparation

For the present study, *Enterococcus faecalis* (ATCC 29212) was grown on chocolate agar (Brain Heart Infusion agar, Oxoid, Basingstoke, UK), for 24 hours at 37°C in CO2. Then, the *Enterococcus faecalis* were delivered into the tubes containing 5 mL sterile BHI (Brain Heart Infusion agar, Oxoid, Basingstoke, UK) suspension, which were adjusted to a turbidity of 1.5 × 108 colony-forming units (CFU)/mL-1. One milliliter of microbial suspensions was placed in the upper chamber of the prepared apparatus. Before bacterial intubation into upper chambers, the specimens’ apparatus were incubated for 24 hours at 37°C in order to evaluate the samples contamination during mounting process. If the BHI medium was turned turbid, it was considered to be contaminated and the previous procedure was repeated. Then, all the glasses were incubated (Fig. 1D) at 37°C for 90 days. After 24 hours incubation, positive and negative controls were checked to ensure the reliability of test. Every three days, 1 mL of the suspension (BHI containing *E. faecalis*) were aspirated from the chamber and replaced by 1 mL of fresh BHI inoculated with *E. faecalis*. The specimens were observed every day for turbidity of the broth in the lower chamber, indicating bacterial growth resulting from penetration of the bacteria past the root canal (Fig. 1E). To confirm that the BHI turbidity was because of the *E. Faecalis* growth, the turbid BHI medium was cultured on a chocolate agar medium with a sterile swab. After incubation for 24 hours, colonies were examined and stained with gram-staining and studied under microscope. Then according to hemolysis type, catalase, bile escullin, PYR, optochin disk and 6.5% NaOCl growth tests were performed. The day of turbidity was recorded.

-Statistical analysis 

Kolmogorov-Smirnov test was used to check normal distribution of the data. Because the obtained data did not have a normal distribution, non-parametric tests were used. Medians and ranges of medians were assessed for the values of descriptive statistical analysis. Leakage comparison between experimental groups was done using Mann-Whitney test by Spss 16 software at significancy level of 0.05.

## Results

In all positive control specimens, lower chamber BHI medium turned turbid at 24 hours after incubation. During examination interval, none of negative controls turned turbid. The *E. faecalis* was isolated from all turbid specimens by means of enterococcal diagnostic tests (since the colonies were α-hemolytic, opthocin disk, bile escullin, and NaOCl growth tests were carried out). The colonies were resistant to optochin disk, bile escullin resistant, and had growth on 6.5% NaOCl medium. Therefore, they were enterococcus species.

**Figure 2:**
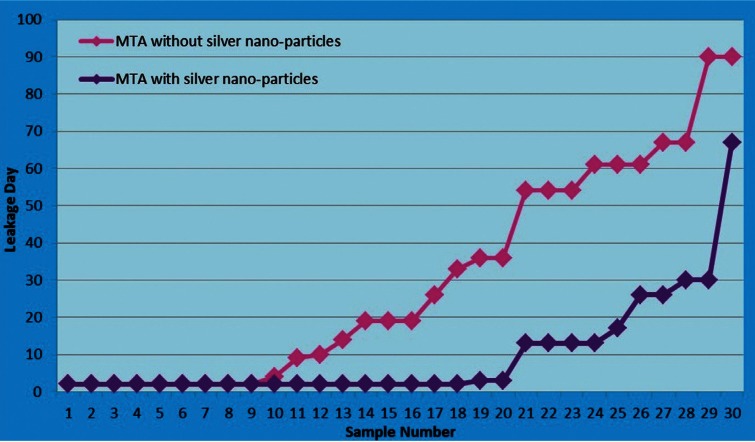
Daily change of bacterial leakage in groups studied.

The median bacterial leakages in experimental samples and ranges and mean bacterial leakage days and standard deviations are shown in  Table 1 and figure 2. The median bacterial leakages for MTA and MTA with nanosilver were 19 and 2, respectively. The mean bacterial leakages for MTA and MTA with nanosilver were 30.06±28.67 and 9.66±14.25, respectively. Mann-Whitney test indicated that there was a significant difference in bacterial leakage time between two experimental groups (*P*=0.002). On the other hand, MTA with nanosilver samples leaked earlier than MTA group. Comparison of the frequency of the leaked samples in three thirty day’s intervals is shown in  Table 2. In the first 30-day interval, 93.33% of MTA with nanosilver samples were leaked but this was 56.66% for MTA group. In the second 30-day interval 3.33% of MTA with nanosilver samples were leaked but this was 20% for MTA group and in the third 30-day interval, 3.33% of MTA with nanosilver samples were leaked but this was 23.33% for MTA group. Although most samples in two groups leaked in first 30-day interval, but this was more in the MTA with nanosilver group.

**Table 1:**
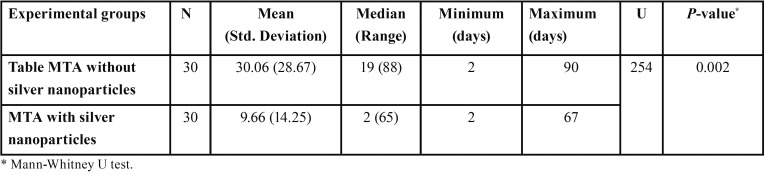
Statistical data of the bacterial leakage of experimental groups.

**Table 2:**
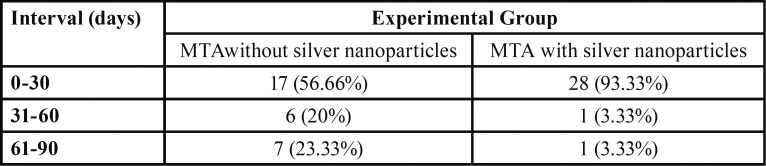
Comparison of the frequency (%) of the samples with bacterial leakage in three studied intervals.

## Discussion

Previous studies have demonstrated that nanosilver enhance some properties of MTA (7,13). However to the best of our knowledge the present study for the first time investigated the effect of nanosilver on sealing ability of the MTA.

Gomes-Filho *et al.* (13) evaluated the tissue response to implanted polyethylene tubes filled with fibrin sponge embedded with nanosilver dispersion. They concluded that nanosilver dispersion was biocompatible, mainly at low concentrations. Therefore, low concentration (1% by weight) and small particles (<150nm) were used in this study to reduce toxicity.

The results of Samiei *et al.* (7) study revealed that adding nanosilver by 1% weight to MTA improve its antimicrobial activity against *E. faecalis*, *C. albicans* and *P. aeruginosa*. Nanosilver ascertained to combine good antibacterial effect in the lack of cytotoxicity (14). The antibacterial effect of nanosilver is due to binding to crucial cellular structure components like enzymes and other proteins, mainly to their SH-groups (15). Silver ions interfere with the integrity of the bacterial cell, its energy production and preservation (5,6,15). This multilevel antimicrobial mode guarantees that resistance cannot be simply acquired by single point mutations in compare with aminoglycoside antibiotics, where resistance can be acquired much easier (14).

Sealing of all pathways between coronal and apical portion of the root canal system is the main objective of root canal therapy and is necessary for its long-term success (10). The most commonly used root end filling material is amalgam but it does not provide a satisfactory seal and there are numerous disadvantageous with this material (16). Sealing ability of MTA has been proved to be superior to that of amalgam or Super-EBA in different dye and bacterial leakage investigations and several studies have indicated that MTA exhibits less leakage in comparison to other materials (17).

Most widely used methods in assessment of the leakage are the dye penetration method (18), electrochemical leakage test (19), and the fluid filtration technique (20). In several studies limitations of the dye penetration method were mentioned. Some authors suggested that air inside the root canal filling may prevent the penetration of dye (21). Camps and Pashley (22) stated that dye penetration relies on randomly cutting the root into two pieces without knowing if the section goes through the deepest dye penetration. The disadvantage of the electrochemical test was stated by Amditis *et al.* (19) that corrosion forming on the anodes blocks the diffusion of ions as well as salivary or extraneous products. Bacterial leakage evaluation is a three-dimensional examination of leakage through the canal system and would better simulate the clinical conditions and bacteria and bacterial products have also been used (23). Therefore, the bacterial leakage test was performed to evaluate the sealing efficacy in this study and this is one of the distinguishing features of the present study. However, leakage studies cannot be repetitive and generalizing these *ex-vivo* results for clinical use must be done with caution. This is clearly clarified by the cold lateral condensation technique which causes abundant leakage *in vitro*, yet has a 90% clinical success rate (17).

Various bacterial species have been used for bacterial penetration analysis. *Enterococcus faecalis* was used in this study because it is the most virulent bacteria in root canal system and is considered to be the predominant bacteria in failure of root canal therapies and chronic apical periodontitis (23). Regardless of bacterial species, the important aspect of this test was to confirm that the positive result was because of the specific bacterial growth and not of contamination during sample preparation. This is another distinguishing feature for present study. In this study we confirmed the positive samples by different isolating tests such as bile escullin, optochin disk, and growth on 6.5% NaOCl medium. Although bacterial leakage method measures the exact penetration of bacteria, this cannot mirror the clinical condition as only one type of bacteria is used (23).

Bortoluzzi *et al.* (24) compared the sealing ability of MTA with and without calcium chloride as a root end filling material by dye leakage approach. They reported that adding calcium chloride to MTA improved its sealing ability. They declared that enhancement in sealing properties by adding calcium chloride would be attributed to acceleration of MTA setting time with calcium chloride which directly decreases material leakage. In contrast, Almeida *et al.* (25) reported that the addition of calcium chloride to the MTA negatively influenced the apical seal. They noted that MTA needs a smaller amount of water for mixture when combined with calcium chloride, leading to changed powder-liquid proportion and increased porosity. Above the ideal proportion, water causes formation of many capillary pores, which increase shrinkage and cracking with consequent loss of sealing ability.

Brito-junior *et al.* (26) demonstrated that the use of propyleneglycol as a vehicle for gray MTA increased its sealing ability in furcal perforations which was attributed to the better homogeneity and decrease in cement prosity. Moreover, the mixture of Propyleneglycol-MTA could have favored a greater setting expansion of MTA, which is one of the possible reasons for the good sealing ability of MTA. Chung *et al.* (27) demonstrated that 4-META/MMA-TBB resin as a mixing vehicle of MTA powder would improve its sealing ability. The short setting time of MTA/4META, and possibly the bonding ability of 4META/MMA-TBB resin to dentin and cementum, would be a plausible explanation for its enhanced ability.

Aruda *et al.* (28) reported significantly better sealing ability for MTA combined with doxycycline compared to MTA mixed with distilled water. This difference can probably be explained by doxycycline properties and apparently shorter setting time and the more friable aspect of MTA plus doxycycline. In a study by Shantiaee *et al.* (29) the apical sealing ability of standard and nano-silver coated gutta-percha as root filling materials was compared by dye and bacterial leakage methods and no difference was obsereved neither in bacterial leakage nor in dye leakage results.

Our findings depicted that MTA without nanosilver exhibited better sealing ability than MTA with silver nanoparticles. The results from present study demonstrated significant difference in bacterial leakage between two groups. Bacterial leakage is more important in early days after obturation of canal because of existing more bacterial contamination in the area. After 30 days, with decreasing nutrients, a gradual decrease in the amount of bacteria is expected. This study indicated that in the first 30-day interval, 93.33% of MTA with nanosilver samples were leaked but this was 56.66% for MTA group. Although most samples in two groups leaked in first 30-day interval, but this was more in the MTA with nanosilver group. In MTA group, most of the samples leaked at day 19 and in MTA with nanosilver group leakage was observed at day 2. This revealed that MTA with nanosilver samples leaked earlier than MTA group.

Some possible explanations for poor sealing ability of MTA with nanosilver could be the disturbed homogeneity of material, disturbance of water absorption, and less expansion during setting. In addition, significantly higher bacterial leakage in the MTA with nanosilver group might be due to its greater solubility after setting. It has been noted that MTA becomes more porous and soluble if the powder-liquid ratio is decreased (30). Furthermore changed powder-liquid proportion could increase shrinkage during setting and cracking and consequently loss of sealing ability. More studies are warranted to investigate underlying reasons of this finding.

## Conclusions

Although previous studies have demonstrated that nanosilver enhance some properties of MTA such as antimicrobial activity but based on the findings of this *in-vitro* bacterial leakage study, adding nanosilver to MTA decreased its sealing ability.
